# Re-examining utopia in contemporary consumption: conceptualization and implications for marketing

**DOI:** 10.1007/s13162-021-00193-0

**Published:** 2021-02-19

**Authors:** Aleksandrina Atanasova

**Affiliations:** School of Management, Royal Holloway University of London, TW20 0EX Egham, England

**Keywords:** Utopia, Liquid consumption, Desire, Escape, Experiential consumption, Retailing

## Abstract

This paper introduces liquid consumer utopias, defined as market-mediated expressions of individuals’ desires to re-imagine and re-construct reality, and to re-frame the present. This conceptual lens illuminates previously untheorized consumption phenomena, which are socially constructed, and often critical, efforts to enact an alternative way of being in an increasingly uncertain and unpredictable world. Three key characteristics of liquid utopias are outlined—immediacy, transience and hyper-individualization––each pointing to liquid consumer utopias’ function to facilitate present-oriented and short-lived re-imaginings of reality. Co-existing alongside the solid and collective utopian consumption of interest to prior research, these emergent forms of liquid consumer utopias articulate a re-imagining of the present (rather than the future), have an emphasis on individual (rather than communal) experiences of betterment, and an orientation toward temporary re-framings of the experienced reality (rather than a pursuit of permanence and long-lasting change). Implications are discussed for retailing, experiential consumption, and consumer self-optimization.

## Introduction

Some consumers are paying a premium to eat airplane food in their homes (Cherney, 2020); others queue for hours in empty airports just to take a “flight to nowhere” on aircrafts that take off and land at the same destination a few hours later (Mzezewa, 2020); and others still are “window swapping” as they “trade places” with strangers while staring at a website offering live sights and sounds from Vancouver to Jakarta (Hynes, 2020). These curious consumption phenomena emerged in 2020––the year that brought one of the largest healthcare pandemics in modern history, halted our quotidian ways of life, and in turn prompted a distinct longing for what can no longer be had. Even prior to this notorious year, however, consumers have been yearning for the seemingly inexplicable. Droves of digitally native youth, for instance, have for years been fetishizing knitting, cookie baking, grandma aesthetics and countryside living, spurring mass Gen Z trends such as “grandmacore”, “cottagecore” and “farmcore”––signposts for nostalgia-ridden communities that, paradoxically, thrive on many of the most popular internet platforms of the day such as TikTok (Slone, 2020). What these phenomena have in common is a desire to live in a world outside the one currently inhabited––in the cottagecore universe, there are no phones pinging constantly with updates, no urgent emails (Slone, 2020); similarly, as one boards a flight to nowhere, a brief moment of familiar normalcy is imagined and inhabited––it is not the destination that one is after, but the process of getting somewhere, even if ultimately that might be nowhere.

While such consumption phenomena are growing in prominence in today’s marketplace, they are largely under-theorized in consumer research. Neither particularly intense, risky, nor painful, this type of consumption is unlike the escapist and extraordinary consumption of interest to past scholarship, where escaping the mundanity of the everyday was achieved via extraordinary experiences such as skydiving (Celsi, Rose & Leigh, 1993), competing in Tough Mudder (Scott, Cayla & Cova, 2017), climbing Everest (Tumbat & Belk, 2011), surfing (Canniford & Shankar 2013), river rafting (Arnould & Price, 1993), attending the Burning Man festival (Kozinets, 2002) or the Mountain Man Rendez-Vous (Belk & Costa, 1998). A flight to nowhere or a staged old-world-aesthetic for a TikTok reel can also hardly be considered transformative experiences or even mundane escapes (Cova, Carù & Cayla, 2018) in their own right, for they are not necessarily defined by a search for self-suspension or a wish to escape from self-awareness (see Cova et al., 2018).

What these consumption episodes elicit, however, is a deep-rooted desire for reality to be different than what it is. Such yearning to repeatedly “measure the life ‘as it is’ by a life as it should be” (Bauman, 2003, p.11) has continuously, across literary and academic disciplines, been converging within the notion of utopia––an ambivalent and nuanced construct at the juncture between hope and desire for something else other than what is presently experienced or had (Levitas, 2013). While colloquially utopia denotes a place that does not exist, or a collectively pursued vision for a perfect world or society, analytically the term is understood to reflect our aspiration toward fulfilling needs that are currently unmet in life as-is—that is, our desire to bridge the implicit scarcity gap between needs and satisfactions (Levitas, 2011). To this end, contemporary utopian scholarship has advanced conceptualizations where utopia is seen not as a descriptive form––a place or an ideology––but as a function with emancipatory potential, a culturally constructed practice whose purpose is to reframe and transform the present (Levitas, 2011, 2013). In today’s consumer culture, consumption is often thought of as the organization and materialization of such utopian practices tasked with transforming one’s lived reality (Bauman, 2007; Fitchett, 2020); it is therefore important that we trace the emergent utopian visions of the day and build the theoretical tools needed to understand their function in shaping consumer behavior.

Drawing from recent advances in utopian theory (Levitas, 2013), as well as theory of liquid consumption (Bardhi & Eckhardt, 2017), this paper conceptualizes the notion of liquid consumer utopias, defined as market-mediated expressions of individuals’ desires to re-imagine and re-construct reality, and to re-frame the present. By integrating critical sociological perspectives on the changing nature of utopia in consumer society (Bauman, 2007), it is theorized that liquid consumer utopias arise as a result of an emergent utopian impulse that sees a re-orientation from solid and communal grand utopian visions to liquid, short-termed and individual utopian pursuits of betterment through everyday consumption practices. We suggest that this utopian impulse motivates a re-imagining of the present (rather than the future), has an emphasis on individual (rather than communal or collective) experiences of betterment, and an orientation toward temporary re-framings of the experienced reality (rather than a pursuit of permanence or long-lasting change). We identify and outline three key characteristics of liquid consumer utopias—immediacy, transience and hyper-individualization––each pointing to liquid consumer utopias’ function to facilitate present-oriented, short-lived and fragmented re-imaginings of reality. Against this theoretical backdrop, this paper advances the notion of liquid utopian consumption phenomena as lower key, yet ambitious, efforts to imagine an alternative way of being, to reshape the day-to-day, and do things otherwise, here and now, albeit for a fleeting moment. In the process of constructing this conceptual lens, we also highlight the context-dependent nature of consumers’ utopian imaginaries and trace how both liquid and solid utopian visions exist in parallel.

Prior consumer research suggests that “the majority of today’s utopias are marketing inflected” (p.679) and that such utopias are to be found within perfect worlds of marketing created dreamlands of abundance and satisfaction such as Disneyland, McDonald’s, Las Vegas, or the shopping mall (Brown, Maclaran & Stevens, 1996; Sherry, 2013). This paper goes beyond this perspective and contributes to a more nuanced understanding of the interface between consumption and utopia as an analytic construct by charting how utopias, in the context of consumption, might be taking new forms––increasingly to be found in liquid and private utopian imaginaries that consumers pursue through resources in the marketplace, rather than in readily discovered dream-like worlds already conjured by the marketplace. Tracing these shifts speaks to an understanding that utopian visions are never arbitrary, but embedded in their socio-cultural, political and economic contexts (Gordin, Tilley & Prakash, 2010; Levitas, 2013). To this end, the proposed theorization of liquid consumer utopias contributes to the literature by offering a lens that illuminates how consumers construct visions for a different and subjectively better way of being in today’s world. Faced with unmanageable natural and geopolitical events, health pandemics and a widespread sense of polarizing global political crisis, many consumers are continuously dealing with a heightened degree of uncertainty in relation to both what is to come and how it may be dealt with once it arrives (Cook, 2018). In such times of uncertainty, visions for betterment energize; theorizing liquid consumer utopias helps us trace how such visions can materialize through various forms of consumption. With that, this paper broadens the conceptual terrain between utopian scholarship and marketing, and extends recent research where efforts to “develop a more systematized understanding of contemporary consumer utopias” (Kozinets, 2019, p.66) have begun to recognize the multi-faceted nature of utopian consumption.

This work advances marketing theory through envisioning (MacInnis, 2011) the elusive construct of utopia in a new, phenomenologically relevant way, which can help us better understand otherwise hidden macro dynamics within the marketplace. In doing so, this paper expands our theoretical arsenal from the margins (Vargo, 2019), drawing on perspectives outside of the discipline’s core and building on the tradition within Consumer Culture Theoretics, wherein theory construction begins with delving into the sociohistorical and sociocultural dynamics that influence the symbolic and ideological aspects of consumption (Arnould & Thompson, 2005). This approach to examining consumption through a culturally grounded, utopian analytical lens allows us to suggest fruitful new avenues for research in marketing; three such avenues are examined in detail in this paper: retailing, experiential consumption, and consumer self-optimization.

Next, we review the theoretical underpinnings and trace the strands of utopian perspectives outside and within consumer research. Then, we map out the conceptual terrain of emergent contemporary utopian imaginaries and theorize liquid consumer utopias. Finally, we discuss the implications of this theorization for marketing.

## Utopia and marketing

### Theoretical underpinnings of utopia as a concept

An enduring fixture in the everyday vernacular, utopias are largely thought of as impossibilities and mere reflections of imaginaries never to be realized (Kumar, 1991; Levitas, 2011). As a scholarly construct, however, utopia is a much more ambivalent conception. This ambivalence has been largely inherited from Sir Thomas More’s 1516 satirical novel *Utopia* whose title alone was intentionally ambiguous––a pun for a good place and no place at once, bounded by its territory and tempting with its promise for finality, the ultimate paradisiacal destination that did not exist, yet beyond which nothing better existed either. The world which More describes is a world of contradictions that can be read either as idealistic or as impractical––a deliberate ambiguity, which has rendered utopia an ideological battleground for literary, political and cultural scholars, anthropologists, sociologists and academics at large (Levitas, 2011). To that end, there are three primary ways in which social theorists have used the term utopia: as defined by their content, form or function (Levitas, 2011, 2013).

First, utopias can be defined in terms of content––that is, what is *a* utopia (Sargisson, 2002). Depending on the historical and sociocultural context, utopias can portray different prescriptive versions of ideal societies or alternative worlds, reflecting issues which appear to be important to different social groups at a specific time. Through this lens, the content of utopia can vary significantly across time and places––past, future, Utopia, Atlantis, Shangri-la, Cockaygne––but the content tends to be evaluative and normative, specifying in detail what a good society would be and how it would function (Levitas, 2011, p.5). Stretching from early modernity to the present day, a canon of utopian—and, latterly, anti-utopian or dystopian—texts has been established to relay various such normative prescriptions of utopia (Garforth, 2009).This points to a second approach to defining utopias––descriptively. That is, in terms of the form through which utopian ideals are expressed. As such, utopia can be defined as a literary genre, imaginative fiction, strands in political theory, or myths (Levitas, 2011). Within this definition too, utopias most often describe what a good society would look like (whether possible or not) and tend to detail normative blueprints of an ideal commonwealth, but the focus is on the different forms and expressions through which this is done. To this end, More’s *Utopia* established what is seen as the quintessential utopian form, which has been replicated across numerous literary pieces where a basic narrative pattern portrays a visitor from another place or time encountering a superior civilization (such portrayals have been generously enhanced by satire, which later would bring about the literary sub-genres of dystopia or anti-utopia) (Kumar, 1991, p.26–27). Like other literary utopian works, More’s utopia depicts a journey (an escape), a voyage toward a world that stands in total contrast with the harsh reality of the day and where no human need is left unattended (Brown et al., 1996).

Inherent to the utopian literary genre is the issue of impossibility relayed through the notion of distance, for these works seek to portray worlds at distinguishable distance from reality. In other utopian forms however, such as utopian socialist writings, the notion of impossibility has been deliberately offset; there, the issue of realization and possibility have been intrinsic to the appeal of the transformational process in overcoming the poverty and degradation that were characteristic for the early industrial society which inspired these writings (Levitas, 2011, p.42). Yet again however, throughout the twentieth century, the notion of utopian impossibility was further reinforced by the multitudes of failed socialist utopias turned dystopias––idealistic visions either gone entirely wrong or functioning only for particular segments of society (Gordin et al., 2010). Overall, on these accounts, prior to the postmodern turn in social and cultural theory, most definitions of utopia have been concerned with either the content or the form of utopia, resulting in a tendency to think of utopia as either a totalitarian political project, or a literary genre of fictions about perfect societies (Levitas, 2012).

In late modernity, however, a new approach to theorizing the utopian imaginary has come to the fore: a marked shift from an emphasis on representation or content (a perfect society, a fictional land) to an emphasis on process (an individual practice seeking to transform the everyday) (Levitas, 2012; Bauman, 2007). In turn, in contemporary utopian scholarship, a third way of defining utopia is in focus––utopia seen in terms of its function, that is, in terms of what it does. This viewpoint culminates in Levitas’ definition of utopia as “the expression of desire for a better way of living and being” (2013, p.4)––a definition which not only isolates *desire* as the shared element among the disparate forms and contents of utopia throughout time (Garforth, 2009), but crucially foregrounds desire as the key in unraveling the function of the utopian imagination. Utopia can thus be conceived as a way of attempting to remedy the experience of lack, of dissatisfaction, of “something’s missing,” in the actuality of human existence, as it unfolds through time (Levitas, 2012)*.* Such a definition is analytic rather than descriptive and it thus enables us to look at the utopian aspects of various cultural forms and expressions (Levitas, 2013).

Through this perspective, utopia may take place in different socially constructed forms (Levitas, 2005): imagining an alternative future is only one such manifestation (consider social justice movements such as Extinction Rebellion or Black Lives Matter, demanding radical structural and sociocultural change); utopia may also transpire within a more personal locus, in the quest for the ideal relationship, or for the perfect self or body (consider the human potential movement of the 1960s and the ensuing—and enduring to this day—interest shown by mainstream society in personal development, self-help, the quality of relationships and emotional literacy; see Puttick, 2000). Thus, while expressions of utopian longings may critique dominant ideologies or explore oppositional ways of living and being, they need not always be necessarily profound (Levitas, 2012)––they can be escape attempts (Cohen & Taylor, 1976) seeking to transform the daily struggle or routine and change one’s place within the world, rather than change the world itself (Levitas, 2011, p.99). Uniformly, however, seen as expression of desire for a better, and thus different, way of living and being, utopia emerges as a form of counter-factual thinking (although not always self-consciously so) (Levitas, 2005, p.198).

The function of such a liberatory mode of thinking is to open up the possibility of apprehending another way of being, one that can be glimpsed from within a dominant social totality (Garforth, 2009). Coincidentally, historical research shows that utopian visions thrive in times when political and social patterns grow tedious or troubling (Friesen & Friesen, 2004). Various utopian imaginaries, for instance, flourished in America in the politically turbulent 1970s in protest against the fetishization of achievement, competition, or materialistic success. The hippie movement and related sub-cultures sprung as an embodiment of antiestablishment behavior––the lived reality was critically reimagined and countered via alternative fashions, belief in enlightenment through drugs, experimenting with unusual sexual behaviors, embracing Eastern spiritualities, astrology, and the like (Friesen & Friesen, 2004; Spencer, 1990). Tracing the function of such liberatory and critical expressions reveals utopia not as a “natural impulse” that is socially mediated, but a socially constructed response to an equally socially constructed gap between needs, wants and satisfactions generated in a society (Levitas, 2011, p.210). We adopt this function-view orientation toward the analytic (rather than descriptive) construct of utopia and we adopt Levitas’ definition of utopia as “the expression of desire for a better way of living and being” (2013, p.4) while we trace the utopian imaginary within contemporary consumption phenomena as it unfolds in our dynamic present-day context.

### Liquid modernity and utopia

Studying utopia in terms of its function positions the construct not just as a conception anchored in a certain space–time coordinate, but as a practice (practice in this sense is understood to mean a nexus of performances, doings and sayings; see Warde, 2005), a lens used by actors for understanding their particular circumstances and by researchers for understanding contemporary culture (Gordin et al., 2010). Late modernity witnesses a contemporary culture that is ridden with generational anxieties and burn-out (Petersen, 2020), existential and ontological insecurities (Areni, 2019), acceleration (Rosa, 2013), and information overload (Hemp, 2009). These trends are epiphenomenal to what Zygmunt Bauman has labeled “liquid modernity” (2000)—the social condition of increased mobility, fluid identities, and weakening of established social norms and institutions within contemporary society. At the heart of the conception of liquid modernity are the notions of change, uncertainty and transience. Utopia has a central place in times of uncertainty and, consequentially, is an integral part of the theory of liquid modernity (Bauman, 2000, 2007).

To that end, it has been suggested that the social and cultural dynamics in late modernity have instigated a transformation in the utopian imaginary: a shift from communal, solid and long-term visions, to individual, liquid and short-lived desires (Bauman, 2003, 2007). Bauman, himself a function-view utopian thinker, has consistently focused on describing this transformation of utopia and the causes and consequences of this shift (Jacobsen, 2008). For him, we live in an era where a new and unseen spirit of liquid utopianism is taking hold (Jacobsen, 2008) in which “the ‘place’ (whether physical or social) has been replaced by the unending sequence of new beginnings […] and the desire of a different today has elbowed out concern with a better tomorrow” (Bauman, 2003, p.24). In developing these ideas, Bauman distinguishes between what he theorizes as solid and liquid utopias. He defines solid utopias as typified by their territoriality and finality—that is, they portray or pursue visions of a better life that are confined to a clearly defined territory and they inhere a potential to reach a natural conclusion at some point where any further change could only be a change for the worse (Bauman, 2003). These solid utopias’ function is to envision and/or enact a desired future; they occur within practices that are collective, holistic, forward-looking and often manifesting themselves in different movements, communities or tribes. Liquid utopias, in contrast, are temporary and highly individualized, embedded within and unfolding alongside the rhythm of contemporary society. Their function is not to seek shared improvement or to envision a desired future, but to creatively transform the present moment through fragmented and individualized action (Bauman, 2003, 2007; Jacobsen, 2008).

While Bauman’s view positions solid utopias firmly in the past, and liquid utopias in the present, we use his theorization not as a blueprint, but as a heuristic device that is useful in eliciting and tracing the shifting trajectories of utopian desire in our present day. To that end, we adopt a perspective which recognizes that both solid and liquid *orientations* within the utopian mode of thinking are a part of the social imaginary in various ways. Consider, for instance, the numerous contemporary subcultures and collectives, such as Burning Man (see Kozinets, 2002) or the hipster communities settling in abandoned Western movie sets, turned ghost towns, and seeking to counter the accelerated and socially disparate realties of mainstream life (Krueger, 2016). In these current sociocultural phenomena, visions for betterment and transformation have a more solid, future-orientation where permanence is desired albeit not achievable; the utopian imaginary is conjured up and pursued collectively within designated locales where it could be articulated freely. We thus advance that both solid and liquid utopias can thrive in liquid modernity’s cast of contradictions that transpire in the everyday—consider climate change deniers and activists, Brexit and Remain, return of the analogue and exponential growth of the digital. Of course, similar dichotomies have always existed. In our late modern context, however, they are infused within the daily discourse—influencing, shifting and motivating behavior.

To that end, Bauman argues that today’s utopias’ primary means of expression is through consumption—the ultimate arena for pursuit of desire (see Bauman, 2007, p.94–110). Other contemporary thinkers agree, proclaiming that “today, the market is the source of utopian aspirations, and consumer culture is where we can realize those dreams” and that “we should distinguish between ‘spectacular’ utopian and dystopian spaces, and ‘mundane’ or ‘everyday’ ones” (Fitchett, 2020, p.55–56). It is indeed this expression of utopia through consumption to which Maclaran and Brown (2001, p.370) also referred to when urging scholars to look for the “newtopias” of the day, thus opening paths for the function-view of utopia within consumer research. Ensuing scholarship, however, had largely focused on the “spectacular” consumer utopian visions and grand utopian scapes, such as shopping malls, festivals and the like, leaving many of consumers’ everyday liquid utopian expressions untheorized. This literature is briefly reviewed next.

### Utopia in consumer research

As a central heuristic tool, the concept of utopia has thus far been mostly used to unravel consumption in “emblematic marketing institutions” with “essentially [u]topian or quasi-utopian function” (Brown et al., 1996, p.676), such as immersive consumption-scapes or collective movements. Two of the earliest empirical studies that turn to a function view of utopia examine a shopping mall in Ireland and illustrate how this consumption-scape––in both its form (as a magical discovery one stumbles upon) and function (as a critique to the established norms of shopping) (Maclaran & Brown, 2001)––can be seen as a space with utopian potential, where utopian meanings are collectively constructed between consumers and retailers (Maclaran & Brown, 2005). In a similar vein, Murtola (2010) also focuses on the utopian function of the shopping mall and describes it as a commercial space attractive to the masses which imitates paradisiacal (and well-guarded) templates of harmony and abundance. In Murtola’s view, the critical potential of utopias that unfold within such commercial-scapes, however, is largely absent; instead, she argues, “utopia has been reduced to an instrument of capital accumulation and turned into a form fitting the confines of commercial consumption” (2010, p.46).

In contrast, Bossy (2014) uses utopia as a lens to understand political consumerism where a network of individual and collective actors politicize the act of buying in order to search and promote other types of consumption. Bossy sees utopia both as discourse and a practice that can enable positive collective action through rejection of the existing society and conception of an alternative world. Such conceptions can be similarly seen in the Star Trek fandom which Kozinets (2001) describes as a closely knit “utopian refuge for the alienated and disenfranchised” (p.71) and a commercially facilitated collective utopia that bonds together the Star Trek fan community. Another type of collective utopia is studied by Chatzidakis, Maclaran and Bradshaw (2012) who conceptualize an Athenian neighborhood, renowned for its anti-capitalist ethos, as a critically charged heterotopian space for utopian praxis where collective action and communal spirit can flourish. Finally, and also rooting their analysis in alternative urban spaces, Hong and Vicdan (2016) study ecovillages as ostensibly utopian spaces where collectively shared utopian ideals are re-imagined based on the social configuration of sustainable lifestyles. Across these studies, utopias are materialized through distinct spacial forms intended for collective enjoyment and bound within physical places, and their expressions echo aspirational and prescriptive visions for what society, or one’s lived experience within it, ought to be.

Utopia is also frequently used as a peripheral construct in consumer research. Within this group of studies, the notion of utopia is evoked to bring richness or clarity to emancipatory or anti-structural consumption phenomena via reference to colloquial ideals of what society could or should look like. Within this group of studies, a plethora of consumption contexts and performances have been identified as utopian or holding utopian potential: from depiction of a quasi-utopian world of democratic togetherness on board cruise ships (Kolberg, 2016); to the Burning Man festival where within a confined space, and during a limited amount of time, escapist utopian visions can unfold within a “youtopia—a good place for me to be myself, and you to be yourself, together” (Kozinets, 2002, p.36); to microfinance and entrepreneurial philanthropy (Bajde, 2013) and fantasy reenactment rendezvous (Belk & Costa, 1998).

Overall, the perspective which these studies share outlines an understanding of utopia as a vision that is implicitly or explicitly communal and shared in character, either in the process of envisioning it or enacting it. Such a vantage point frames the issue of desire in relation to spacial forms and engagement with such spaces and/or at the level of community or subcultures of consumption. Since much of consumer research has been notably interested in collective consumption experiences, tribes, flagship marketplaces, and phenomena generally unfolding at the meso-level of analysis (e.g. Belk & Costa, 1998; Kozinets, 2001, 2002; O’Guinn & Belk, 1989), it is not surprising that utopia’s communal nature has often been a useful backdrop for many of these studies. See Table 1 for a review of selected universally recognized literary and political utopian writings, as well as for an overview of the various ways utopia has been defined and used in consumer research.

**Table 1:** Conceptualizations of utopia

Author	Conceptualizations of Utopia	Context
SELECTED LITERARY UTOPIAS		
Plato 375 BC	A utopian state model depicting the political structure, demographics, theology and laws of two ideal cities: the first truly just but austere, the second “feverish” and “luxurious”	Ideal commonwealth
Unknown c.1250	Land of Cockaygne: a medieval folk poem depicting an imaginary conflict-free and deprivation-free land of material abundance, where finest wines flow plentifully in rivers, houses are made of delicacies, and plump geese roast by themselves	Mythical place
More 1516	An ideal welfare state where there is no private property, people are distributed in equal numbers across towns and households, wearing simple clothes, performing essential trades, and dining in communal spaces together	Ideal commonwealth
Bellamy 1888	A depiction of an ideal twentieth century centralized socialist society in the US, where poverty is eradicated, industry is nationalized, and capitalism has been replaced by centralized organizations of production and consumption	Ideal commonwealth
Morris 1890	An alternative to Bellamy’s ideal social state and a counter-view of socialism that is depicting England in the twenty-second century, where money has been abolished and one simply asks for what one wants, craftwork has pushed aside “wage slavery,” contracts of marriage have been replaced by flexible bonds of affection, and industrialism has given way to informal patterns of co-operation. The central theme of this model of socialism is of work as pleasure, subject not to massive centralization but active participation of individuals (see Levitas, 2011)	Ideal commonwealth
Wells 1905	Two travelers fall into a space-warp and suddenly find themselves upon a Utopian Earth controlled by a single World Government, where all people share a common language, there is sexual, economic and racial equality, and society is ruled by socialist ideals enforced by an austere, voluntary elite: the “Samurai”	Ideal commonwealth
SELECTED POLITICAL UTOPIAS		
Owen 1813	Promoted a radically different society brought to existence through the setting up of small self-supporting experimental communities where workers were paid what they were worth and shared, rather than competed, in all areas of life. Residents believed that the three main evils of society are religion, private property and marriage	Ideal commonwealth
Saint-Simon 1817	Advanced the ideology of “industrialism” and believed that industrial development provides the conditions for abundance and elimination of poverty and ignorance. Envisioned holistic transformation of the whole society (rather than gradual change in small experimental communities)	Ideal commonwealth
Fourier 1830	Promoted a form of ideal society comprised of small, self-supporting and primarily agricultural communities, called “phalanstères,” where choice of occupation, congenial company and variety of the work itself would mean that labour would cease to be an imposition (see Levitas, 2011)	Ideal commonwealth
CONSUMER UTOPIAS		
Maclaran and Brown 2001	“[A] significant part of western culture [...] ) that provides a crucial critical function for engaging with reality and for perpetually rearranging one’s place in that reality [...] ” (p.369)	Shopping mall
Kozinets 2001	Implied: “[A] world without injustice, intolerance, or poverty” (p.73)	Star Trek convention
Kozinets 2002	Implied: “[E]xperience of caring human contact in a society ‘whose economic and technological dynamic attrits and intrudes upon the integrity of the cultural process’ (Harvey, 1997)” (p.21)	Burning Man festival
Maclaran and Brown 2005	“Not just an idyllic place [but] an activity, a trajectory, a process”; “a heuristic device for perfectibility”; “a process that draws attention to the gap between what it is and what could be” (p.312)	Shopping mall
Murtola 2010	“Utopia is, in this sense, the eternal not-yet-here but potentially to come, and simultaneously also the potentially not to come. As such, it is the ever-new, the desired future that always eludes the present” (p.38)	Shopping mall
Chatzidakis, Maclaran and Bradshaw 2012	"Whereas the concept of utopia envisages a future state of perfection, heterotopia is in the here and now, facilitating [...] the function or process of utopian thinking rather than its ultimate realization […].”; “Heterotopias [...] are collective or shared spaces of ‘otherness’ where alternative forms of social organisation take place, forms that stand in stark contrast to their surrounding environment" (p.497)	Radical Athenian neighborhood
Bossy 2014	“A discourse and a set of practices. The utopian discourse includes, first, a rejection of the existing society, and, second, if not a clear conception of what another world might look like, at least the idea that another society is possible and desirable. The utopian practices need to be an attempt to create here and now at least some of the features of this utopian discourse in the hope of a spread in the rest of society” (p.179)	Slow food movement
Roux 2014	“[U]topias are places that are nowhere (Maclaran & Brown, 2005), sites with no real place (Foucault, 2009), ‘spaces that are fundamentally and essentially unreal’ (Foucault, 1967/2001, p.1574), but whose purpose is to remedy the deficiencies of the present world (Kozinets, 2001)” (p.62)	Tattooing
Kolberg 2016	“Impulse and longing for the dissolution of class striation” (p.6)	Carnival Cruise
Hong and Victan 2016	Emancipatory and reactive, “a very modern phenomenon that considers spatial enlightenment a societal-level priority” (p.121)	Eco-villages
Kozinets 2019	“Utopianism: The attempt to create significantly better societies by first challenging dominant social institutions, such as capitalism, socialism, contemporary politics, or communism Metopianism: A self-oriented view of consumer utopia; a conception of consumers' views of world betterment as embedded in marketing and consumption-based improvements in practices, goods, and services, leading to a personal experience of ever-improving standards of living Wetopianism: A collectively oriented formulation of consumer utopia; a conception of consumers' views of world betterment as based on a questioning of, and challenge to, extant and embedded industrial systems of marketing and consumption that might hide social inequities and ecological consequences” (p.69)	YouTube clicktivism
Roux and Belk 2019	“Utopia […] projects imagination into a better elsewhere [which] provides idealists with romantic desires of transformation that challenge reality” (p.486)	Tattooing

As alluded to earlier, however, in liquid modernity, loss of stable social structures, withering of long-term thinking and planning, alongside gradual withdrawal from collective action and social solidarity, are pushing the nature of utopia and its emancipatory function away from its collectivist origins, toward a realm of hyper-individualization and commoditization (Bauman, 2003, 2007). To capture this, this paper advocates for an expanded theorization of the nexus between utopia and consumption; a theorization that allows that utopia may be fragmentary, fleeting, elusive, with its primary function being to disrupt the taken-for-granted nature of the present (Levitas, 2013). Using such a lens can help us better understand how consumers use consumption to navigate the multitudes of tensions and circumstances in our contemporary times and envision a better way of being in the world.

In recent consumer scholarship, we have already begun to see interest in exploring the notion of individualized utopias: in conceptual (Roux, 2014) and empirical (Roux & Belk, 2019) work on tattooed bodies as sources of utopias and as “places” where “embodied heterotopias” can be produced; as well as in empirical work on heterotopian selfie practices (Rokka & Canniford, 2016). Improving our theoretical grasp of alternative, individualized utopian imaginaries is therefore timely. Importantly, this is not to say that utopia at the level of the collective is no longer analytically relevant: as Kozinets (2019) has recently shown with his work on YouTube utopian clicktivism, collective utopian visions that elicit shared focus on common goals continue to have a role in our contemporary discourse. As such, liquid and solid utopian orientations co-exist within the contemporary consumption landscape. The theorization offered here, however, seeks to shine light on emergent forms of utopian consumption that have thus far been left out of sight. We construct this conceptual lens next.

## Liquid consumer utopias

Proposing that both the finality and territoriality of earlier solid utopian projects are becoming problematic in liquidity, Bauman reads the resulting change as a privatization of imagination in the utopian impulse––a shift from the collective to the individual, from structures to experience, and from a distant future to here and now (Levitas, 2003). His argument about these changing contents of utopian desire develops largely against the backdrop of his understanding that consumption, under the disguise of pursuit of happiness, has emerged as the only achievable utopia in our contemporary consumer culture. He thus sees contemporary utopias as very much active, but also private endeavors that are “cut to the measure of ‘individualized society’” (Bauman in Rojek, 2004, p.309). In liquid modernity, Bauman sees consumption as conjugated to desire and to desire’s even more liquid form––the wish (Lee, 2005). His analysis of consumption takes the experiences of wish fulfillment to be the epitome of discrete utopian actions accomplished without involvement of others (Lee, 2005). Utopias in the context of consumption therefore emerge as desires and wishes for alternative ways of living and being in the world that are expressed and pursued through private acts of consumption. We use this orientation to conceptually outline the shift within utopian thinking as it relates to consumption.

At the meso level, we conceptualize the notion of liquid consumer utopias, defined as market-mediated expressions of individuals’ desires to re-imagine and re-construct reality, and to re-frame the present. Drawing on Bauman (2007), we theorize that liquid consumer utopias arise as a result of an emergent utopian impulse that sees a re-orientation from solid, communal and conditionally distant utopian visions to liquid, short-termed and individually enacted utopian desires for betterment. As such, we propose that liquid consumer utopias articulate a re-imagining of the present (rather than the future), have an emphasis on individual (rather than communal or collective) experiences of betterment, and an orientation toward temporary re-framings of the experienced reality (rather than a pursuit of permanence and long-lasting change).

The utopian imaginary within consumption, however, is necessarily reflective of the broader context and immediate circumstances which waver in the unpredictability of late modernity. In turn, we suggest that consumption can be framed by either solid or liquid orientations within a utopian mode of thinking and that both solid and liquid consumer utopias can co-exist within contemporary consumer culture (see also Bardhi & Eckhardt, 2017). To that end, we propose that solid consumer utopias emerge when ideals of collective betterment and communal experiences are sought after, and/or when the yearning for transformation is concerned with grander visions for society and one’s place in it. Solid utopias are propelled by desire for the transformation to be long lasting and a belief that the upcoming change for the better is, or at least could be, in the offing. See Table 2 for a comparison between solid consumer utopias and liquid consumer utopias.

**Table 2:** Characteristics of solid and liquid consumer utopias

Solid Consumer Utopias	Liquid Consumer Utopias
*Attributes*	*Attributes*
DISTANT & FUTURE-ORIENTED	IMMEDIATE & PRESENT-ORIENTED
Conditionally distant and based on a belief that the desired future will eventually come	Prompted by uncertainty for what is to come and focused on transforming the present
LONG TERM & PERMANENT	SHORT-LIVED & TEMPORARY
Oriented toward long-term planning and desire for the transformation to be long lasting	Oriented toward short-term pursuits of individualized desires and fulfillment of ephemeral visions
COLLECTIVE & HOLISTIC	HYPER-INDIVIDUALIZED & FRAGMENTED
Undertaken or experienced by collectives/groups in pursuit of holistic betterment and change	Undertaken at the individual-level and manifesting as fragmented episodes of betterment and change
*Example Consumption Contexts*	*Example Consumption Contexts*
Festivals and group consumption (e.g. Burning Man; Kozinets, 2002)	Relationships between people and places / things (e.g. decluttering and minimalism; Kondo, 2014)
Grand physical retail spaces (e.g. shopping malls; Maclaran & Brown, 2005)	Individual consumption at home (e.g. Netflix and chill; Young, 2016)
Socio-political groups and urban communities (e.g. eco-villages; Chatzidakis, Maclaran, & Bradshaw, 2012)	Mobile consumer collectives / networks (e.g. digital nomads; Atanasova & Eckhardt, 2021)
Consumer collectives, fandoms and brand tribes (e.g. Star Trek; Kozinets, 2001)	Identity projects / the body / optimization of the self (e.g. self-quantification, tattooing; Roux, 2014)

Within this theoretical lens, we advance the notion of liquid consumer utopias as lower key, fast paced, and individually enacted efforts to imagine an alternative way of being, to reshape the day-to-day, and do things otherwise, here and now, albeit for a fleeting moment. Several features define the nature of such liquid utopias in today’s context: 1) they are immediate and present-oriented, propelled by an instant gratification mentality (Jacobsen, 2008); 2) they are short-lived, resembling “hunts” (Bauman, 2007) performed by utopian hunters, “sensation seekers” that are constantly searching for fulfillment of desires through consumption; 3) and they are highly fragmented and individualized, reflecting a transition from a discourse of shared improvement to that of individual survival (Bauman, 2007). These attributes are synthesized here within three key characteristics of liquid consumer utopias: immediacy, transience and hyper-individualization.

### Immediacy

First, unlike solid utopias, whose function is to envision or enact a desired future, liquid consumer utopias enable transformation of the present through their immediacy. They are lived, rather than being lived towards (Bauman, 2007). As such, liquid utopias are anchored in the present, propelled by an all-encompassing fear of missing out which fuels an eager pursuit of instant gratification in a race against the speed of our everyday. These utopian visions thrive in the consumer society of liquid modernity, where for many, life emerges as a daily market-mediated cycle for developing and fulfilling desires and wishes (Blackshaw, 2005). Momentary and immediate, liquid consumer utopias manifest as fleeting moments of satisfaction and relief. In the contemporary marketplace, access-based consumption (Bardhi & Eckhardt, 2012), for instance, through its quick cycles of acquisition and disposal, is particularly conducive for the immediate realization of consumers’ liquid utopian imaginaries. With its limitless potential for instantaneous consumption, access-based consumption enables consumers to have and experience virtually anything, here and now, without the burdens of ownership nor its demands and prerequisites. Consider the offerings of Rent the Runway or AirBnB Luxe for consumers eager to re-imagine their not so affluent reality and seeking to circumvent its limitations––a different present no longer needs to be merely fantasized about; one could actually visit and observe it, and some of us could even acquire it, albeit for an instant (Fitchett, 2020). In liquidity, whatever might the contents of one’s utopian desires be, they always belong to the realm of the possible. Blackshaw (2005) summarizes Bauman’s vision succinctly: “it is the instantaneity of consumer culture and its ability to ‘take the waiting out of wanting’ in delivering *homo consumens*’ hopes and dreams that is today what is imagined as the measure of the success of a life worth living” (p.114). Importantly, however, liquid utopias, although propagated by the market, do not passively reside in the mundane; to be utopian, consumption must inhere a confrontation with commonsense (Bauman in Jacobsen, 2016) and be motivated by an active pursuit of re-framing and imagining life otherwise. The function of liquid consumer utopias is therefore concerned with creatively, and sometimes critically, transforming the present in the moment, via resources in the marketplace.

### Transience

Second, liquid consumer utopias are short-term and short-lived, defined by their transience. In Bauman’s writings, liquid utopias are metaphorically presented as “hunts” performed by individuals who are “constantly looking for prey and for that extra supply of sensation or stimulus to saturate, however unsuccessfully or short-lived, their insatiable appetite for ever more” (Jacobsen, 2008, p.220). Similarly, liquid consumer utopias are not about satisfying articulated consumer needs, but about catering to ephemeral desires in a contemporary society starved for time (see Husemann & Eckhardt, 2019). Consider for instance, the surprising demand for the “flights to nowhere” mentioned earlier, where in an effort to transform and reframe their lived reality during coronavirus lockdowns, deprived travelers compete for a chance to be given a fake itinerary, check in, go through passport control, security, and even board and interact with flight attendants on the aircraft––one that is never intended to take off or at best would take off and land at the same point of departure a few hours later (Wang, 2020).

In liquid modernity, liquid consumer utopias emerge in chasing after and stringing together such short moments of satisfaction. This is a substantively different orientation than that inherent to solid utopian imaginaries, where the desire to transform reality involves a certain hope for permanence and longevity of the transformation (consider the very much utopian “Make America Great Again” movement and the solid aspirations that it embodies). In contrast, a liquid utopian orientation promotes disengagement rather than life-long loyalty, movement rather than rootedness, gigs rather than a career, escaping rather than committing, experiencing rather than accumulating. It exploits and valorizes consumer society's never ending search for stimulation and novelty. Thus, for such sensation-seeking consumer-hunters, “utopia is a utopia of time coupled with a utopia of speed––of time as an episodic and endless series of consumer sensations with no conceivable or coveted end-point in which the only thing that counts is the speed with which to obtain, live through and consume these sensations” (Jacobsen, 2008, p.221). The implication of that in terms of consumption is two-fold: on the one hand, liquid consumer utopias’ transient character opens up possibilities for continuous transformation of the present time and time again, as each new utopian pursuit is charged with potential; on the other, liquid utopias’ ability to satisfy consumer desire is short lived. In their role as episodic pursuits of betterment, liquid consumer utopias thus emerge as means to cope with the ephemerality and temporality that are characteristic for liquid consumption, where value is markedly transitory and context dependent (Bardhi & Eckhardt, 2017). As such, liquid consumer utopias are not exclusive to the elite; for many, they emerge in response to not having the means to access solidity and security, even if it is desired.

### Hyper-individualization

Finally, liquid consumer utopias are defined by their fragmentation and hyper-individualization. In liquidity, the utopian pursuit is in the singular, subjectively constructed and deeply personalized; “unlike the utopian model of the good life, happiness is thought of as an aim to be pursued individually, and as a series of happy moments succeeding each other––not as a steady state” (Bauman, 2002, p.240). As such, liquid consumer utopias’ function is transformative, escapist and emancipatory at the micro-level. They reframe the present without seeking lasting or collective transformation––such would be beyond the scope of liquid utopias. Rather, for the yearning consumer, a liquid utopian lens reshapes the everyday in the singular. Consider, for instance, Netflix binge watching labeled as self-care or glamorizing “staying-in” as “the new going out”; as Young (2016) asserts, “why risk a restaurant when you can order Seamless or sauté premade gnocchi from Blue Apron? Why go to a bar when you can swipe right? Why go to a reading when you can download a podcast?” Liquid consumer utopias, as market-mediated expressions of individuals’ desires to re-imagine and re-construct reality, thus emerge as fragmented, open-ended and hyper-individualized imaginings. Such are born out of individuals’ own privatized pursuits and critical evaluation of their lived reality, and hence no two utopian visions need to be the same. Of course, as Jacobsen (2008) paraphrases, in the hunting liquid utopias of today, most times people hunt alone, but sometimes hunting in packs appears more rewarding and assuring, as when groups desire identical consumer goods and create short-lived and shallow “imagined communities” in order to exclusively claim and obtain them (Bauman, 1992, p.xix). This can be seen in the emergence of various fluid community-enabled marketplace systems that center around utopian ideals and offer alternative ways of being in the world, such as peer-to-peer clothing rental communities (Albinsson & Perera, 2018) and hybrid co-working/co-living residence collectives (Gandini, 2015).

### Solid and liquid utopias in an age of uncertainty

Liquidity is, of course, a metaphor, enabling us to see and analyze the world through a specific lens which is inevitably selective, leaving out much of what might be within view for the sake of a sharper focus on what might otherwise be missed (Lakoff & Johnson, 1981). As such, a liquid lens does not deny the possibility for solidity in utopian consumption nor does it suggest contemporary consumer culture has dissolved into boundless fluidity for everyone everywhere––the solid, which encompasses structure, still very much exists (Eckhardt & Bardhi, 2020b). To that end, it is useful to think about solid and liquid as two ends of a continuum, highlighting that there are middle points combining liquid and solid (Bardhi & Eckhardt, 2017). Prior scholarship has shown that how and why consumers move along such a solid–liquid continuum is dependent on a number of antecedents such as extent of professional and economic scarcity, access to mobility systems, consumers' innate characteristic and others (see Bardhi & Eckhardt, 2017; Lamberton & Goldsmith, 2020). In a similar vein, solid and liquid utopian orientations should also be thought of as co-existing, and reflective of how consumers globally navigate and integrate a realm between solid and liquid consumption, practices, or circumstances.

This is particularly evident in the context of the global Covid-19 health pandemic which has illustrated some of the tensions between solid and liquid. In liquidity, notions of choice, individualization and acceleration are dominant logics that structure everyday life (Eckhardt, 2020). Covid-19 has largely taken away the liberties and choices we have been so accustomed to and it has, in many ways, halted the acceleration inherent to liquidity. For instance, while, for better or worse, the pandemic has forced families to spend more time together (McCracken, 2020) and has sparked a renaissance of sorts to notions of familial togetherness and closeness, it has also rendered such notions deeply problematic, for togetherness has become a vector for the disease, notwithstanding plexiglass screens, face masks and shields. Consequentially, hallmarks of liquid utopian individualism such as introspection, me-time and self-care are being glamorized and becoming even more mainstream than before (Silva, 2017). Moreover, while in this moment of living in uncertainty, many are moving back home, leaving dense cities for more open spaces, and swapping costly urban residencies for more affordable ones, others are leaning into the precarity rather than trying to redress it by opting for van-living instead of taking up a permanent residence with monthly rent (Tsapovsky, 2020). Thus, even in the context of the coronavirus pandemic, which is amplifying needs for safety and stability, many who are facing job insecurity and mounting expenses are pushed to seek more liquid and flexible ways of living.

In parallel however, the pandemic has also challenged notions of lightness. In a consumer culture where minimalism and getting rid of domestic clutter are signals of privilege and affluence from those who don’t have to worry about what unforeseen wants or needs might lie ahead, scarcity of basic goods and empty grocery store shelves have a way of challenging or even reversing such logics, prompting some to overstock their pantries or re-order the very same board games and casual diversions they had parted with, Mari Kondo-style, back when their lives were busier and the boxes were taking up space in a closet (Mull, 2020a). Thus, while the pandemic has brought more solid orientations such as accumulation, as well as solid structures such as government intervention programs and border closings, it has also, in many ways, reinforced the fragmentation, isolation and uncertainty of liquidity (Eckhardt, 2020). Long-term planning or thinking of the future is particularly challenged, for the future is acutely uncertain under the menace of not only the ongoing pandemic but the potentiality of other similar ones to come. This has opened up avenues for various expressions of liquid utopian desire for a different way of being in the world to unfold––through once obscure, now in vogue avocations such as gardening, baking, and the like, consumers are seeking ways to ground themselves in the present, to summon a sense of control over the unpredictable, and to re-frame their lived lockdown realities. 

Theorizing liquid consumer utopias thus does not suggest that solid utopian visions no longer exist, but it allows us to see and explain new consumption patterns, behaviors and dispositions that were not visible before, not only in the broad context of contemporary consumer culture but also in the present pandemic context where the instability and fragmentation of liquidity are particularly pronounced (Eckhardt, 2020). We discuss the emergence of liquid consumer utopias in today’s marketplace next.

## Liquid utopias in contemporary consumption

In contemporary consumption, liquid utopias, with their potential to transform the present, are emergent in a variety of consumption phenomena. If several decades ago a vision of the good life would have depicted a lifelong career, financial stability, secure retirement, and a nuclear family settled into an owned home, today, these are not dreams that everyone aspires to anymore. If before, consumers sought refuge from the mundane reality in festivals, brandscapes and shopping malls, where collective experiences could soften the lonely and fragmented nature of the postmodern context (Maclaran & Brown, 2001), today increasingly more consumers “stay-in” and “opt-out”, avoid brand signification, show a growing distaste for broadcasted tribe allegiance, declutter, and seek extreme personalization from what they consume and experience (Harris, 2017; Rosenbaum et al., 2019). What emerges through this lens is a type of liquid utopian orientation that is grounded in consumers’ perpetual hunt for positive sensations and control. It is an orientation which is adopted systematically and opportunistically––it empowers and fuels the enactment of transformative imaginaries through various resources in the marketplace.

We have already begun to see the implications of the emergent influence of liquid utopian perspectives in shaping global trends. For instance, the phenomenon of downshifting, propelled by the soaring popularity of the Mari Kondo brand, whose ethos is spreading the “life changing magic of tidying up” (2014), emerges as a prime example of a liquid utopian vision materialized in practice. As Kondo is showing consumers how to seek immediate bliss and overall life improvement through mindfulness about the materiality that surrounds them, the hoarders’ cluttered lives are quickly transformed into neat and open spaces inviting abundant possibilities for happiness. The process is easy and instantaneous—all that it takes is a few piles of clothes soon to be disposed of and one is granted happiness here and now, no spiritual enlightenment required. Of course, chances are that the bliss will be short-term, as true emancipation from the market is not possible (cf. Kozinets, 2002). Nonetheless, as the popularity of Kondo demonstrates, through pursuing short-lived transformative practices for betterment, one can simultaneously cater to desires for a better life and relieve some of the burdens imposed by contemporary consumer culture.

Anticipatory visions and desires for the good life also materialize through alternative forms of liquid consumption where access-based consumption is transforming the boundaries of established social codes of conduct. Consider Japan’s Rent-a-Family industry (Batuman, 2018), for people who are short on relatives and need to hire a husband, a mother, or an entire family clan for weddings, funerals or graduations. Immediate, short-term and highly individualized, this consumption phenomenon offers instantaneous solutions for a range of woes: the service is equally useful for single (often career-oriented) women with marriage-obsessed parents who rent fake boyfriends or fiancés, as well as for bachelors who rent wives and children in order to experience having the kind of nuclear family seen on TV. In confronting commonsense and relishing in the idea of having, but not the having itself, this practice foregrounds liquid utopian visions’ potential to transform the present instantaneously through consumption and to materialize an alternative reality albeit only for a short moment.

Similarly, through a liquid utopian theoretical lens, we can begin to see that the Millennial generation’s famed propensity to stay-in and construct the good life away from established norms and socio-economic structures (cf. Harris, 2017) is not a generational idiosyncrasy but in fact a critical liquid utopian impulse—immediate and short-lived. Such an impulse materializes through clusters of consumption practices instilling a sense of making it in the world, even if on a micro, hyper-individualized scale; for example, collecting Instagram-worthy high-end cookware as “trophies of domesticity” and markers of adult achievement, in lieu of traditional markers such as home ownership (Mull, 2020b). Such practices allow this cohort to reframe the narrative of overall “hopelessness” (Chung, 2017) that stems from their relative low purchasing power, toxic competitiveness, instability and dismal job prospects (cf. Petersen, 2020). Thus, Millennials’ liquid utopian consumption practices inhere a sense of control, prioritization of the self, and wellness—fulfilled visions for a better way of being in a world where much is lacking.

The emergent phenomenon in China known as "sang culture" vividly exemplifies liquid utopia’s reframing and transforming potential. Sang—a term that loosely translates to feeling hopeless, demotivated or dispirited—refers to a kind of youth subculture of quiet rebellion and ironic defeatism in an authoritarian regime notorious for its control (Chung, 2017). Sang culture represents more broadly the millennial propensity to “opt out” and push back against traditional values. This is motivated by this cohort’s circumstantial inability to conform to such values (e.g. buying a house, marry when it customary to do so). Consumption is the medium through which sang culture is expressed and disseminated, and the act of opting out is often manifested via consumption of sang-inspired products. One such product, for instance, is Sung Tea– a brand that sells beverages like “my-ex ‘s-life-is-better-than-mine fruit tea”, “can’t-afford-a-house macchiato” and “achieved-absolutely-nothing black tea”. Similarly, the opting-out Millennial in China can also choose products from other brands such as “fat-free and aspiration-free” “Hopeless Drinking Yoghurt” (Chung, 2017). Through deliberate and public consumption of such products, liquid utopian expressions of the desire for a different way of being in the world emerge as momentary bursts of vocalized discontent. These utopian consumption episodes lay in pockets of transformational re-framings of a mundane life that is far away from the one actually desired. Nonetheless, through engaging in sang, a better life is temporarily conceived as possible, or is at least acted upon.

Notably, liquid utopian consumption manifests in lifestyle choices that seek to resolve the tension from living in a bleak present. This is acutely exemplified by digital nomadism—a lifestyle migration phenomenon where cohorts of demographically diverse consumers (from struggling graduates to entrepreneurs and even retirees) choose to let go of most of their possessions and serially relocate, looking for affordable, yet exotic, places to live and building lifestyles outside of traditional work-life structures, homeownership or traditional notions of “success” (O’Reilly & Benson, 2016). Empirical evidence from the digital nomadic context speaks to nomads’ deliberate pursuit of critically oriented “liquid” lives, where the emotional and physical burdens of a 9–5 lifestyle are suspended and counteracted with short-term pursuits of happiness via global mobility and rejection of normativity (Atanasova & Eckhardt, 2021). The good life is pursued and achieved, here and now, through deliberate “lifestyle design” intended to propel the nomad among the “new rich” of our modernity—those with ample time and resources, not necessarily wealth (Ferriss, 2009). Through the conceptualization of liquid consumer utopias we can begin to see that digital nomads engage in hyper-individualized pursuits of utopian desire toward a better way of being and living in the world, where the “hunt” for the next best place and experience is insatiable, and where their critique of rigid, solid structures evokes an urge for a perpetual escape from the constraints of societal expectations. A liquid utopian lens allows us to better understand such consumption desires and motivations—geared toward experiences and short-lived indulgence (not possessions), immediacy (not long-term benefit), and transformative lifestyle experiences considered desirable for they lie outside the margins and solid structures of mainstream society.

The vantage point of liquid consumer utopias presented here, provides a foundation from which particular acts of consumption can be read as culturally variable, socially constructed, and often critical, expressions of utopian desire to re-imagine and re-construct reality, and to re-frame the present. This perspective illuminates utopias as widely at work in everyday life, and often inconspicuously embedded in consumption contexts which may not present or advertise themselves as utopian (Levitas, 2007, 2017). Next, we discuss implications and future research for several domains within marketing.

## Implications for marketing

Theorizing utopian desire through a liquid lens allows marketers to decode some of consumers’ contemporary utopian propensities and to draw managerial insights with an apt analytical focus. The conception of liquid consumer utopias illuminates emergent intersections of marketplace dynamics and consumption in a number of domains in consumer research, three of which will be explored here for their frequent association with utopian notions: retailing, experiential and anti-structural consumption, and consumer self-optimization.

First, given that the retail context is frequently evoked in extant marketing research on utopias, a liquid utopian conceptualization holds potential for opening up new perspectives to studying retail in an increasingly digitalized and dematerialized world. Traditionally, planned retail spaces, such as malls and department stores, have been positioned as quintessential sites for utopian realization, where “the utopian conceptions of consumers and marketers meld” (Maclaran & Brown, 2005, p.312). At its very genesis, the contemporary shopping mall has been envisioned as “the nucleus of a utopian experiment” and a space where “shoppers will be so bedazzled by a store’s surroundings that they will be drawn––unconsciously, continually to shop in a master-planned, mixed-use community” (Gruen in Scharoun, 2014, p.1). Liquid consumer utopias, however, illuminate that many consumers increasingly prioritize hyper-individualized and instantaneous means for catering to their desires which are not easily facilitated by traditional retail environments. In liquidity, such environments are perceived as disseminating mostly mass-produced commercial goods, available to the many, at a time when consumers show a growing distaste for logo-driven brands and mass, pre-packaged offerings (Eckhardt & Bardhi, 2020a).

Living the good life is instead increasingly pursued in a marketplace where ownership centrality is waning (Lamberton & Goldsmith, 2020) and where materialistic orientations increasingly transpire in liquid forms of consumption such as access, sharing or curation of individualized experiences, not possessions, as signals of status and image (Atanasova & Eckhardt, 2021). This suggests that the role of traditional retail spaces as consumerist utopias is rapidly diminishing. The growing popularity of non-traditional types of retail such as pop-up spaces, nostalgia-fueled media themed diners (e.g., Saved by the Bell, 90210, and Die Hard pop-ups) or Instagramable environments like the Ice Cream Museum in New York City, illustrate consumers’ increasing desire for ever-new and ephemeral utopian escapes. We can see this shift in what has been termed a “retail apocalypse,” evidenced by the nearly ten thousand malls and department stores closing in the U.S. alone in 2019 (Peterson, 2019). In addition, even experiential retailers (e.g. Apple, Tesla) have not been successful in driving more in-store traffic or making going to the mall any more exciting (Thomas, 2019). The liquid utopian lens offered here suggests that the much-publicized demise of the once idealized shopping mall reveals itself not only as a casualty of e-commerce, but also as a victim of consumers’ increasing reorientation from solid to liquid in their pursuit of a better way of being and living.

For retailing scholars, this suggests that envisioning malls as paradisiacal utopias might no longer be as useful. Rather, accounting for the changing landscape of the world of retailing (see Roggeveen & Sethuraman, 2020), a liquid utopian approach suggests reconstructing digital and brick and mortar retail as high-speed channels for immersive, efficient and personalized brand-to-consumer (e.g. NIKEiD) or consumer-to-consumer interactions (e.g. Rent the Runway, StockX). Our theorization foregrounds consumers’ fear of missing out and their desire for speedy consumption that can deliver on their individualized visions quickly. This perspective explains why ephemeral retailing formats, where stores rotate in and out frequently throughout the year on short-term leases, are showing promising results in an overall bleak retailing landscape (Thomas, 2019).

Further, AI and VR augmented environments (Heller et al., 2019) that bridge the offline and online are emerging as new retail utopias for consumers’ preference for immediate, transitory and individualized interactions. Consider, for instance, Lululemon’s acquisition of “Mirror” in the aftermath of rapidly declining retail sales as a result of the coronavirus pandemic. Mirror is an exercise hardware startup that has brought to the market an innovative at-home, live-stream reflective display with a built-in camera and speakers which allows users to simultaneously stream workouts while watching themselves to benchmark their performance against an instructor; from boxing to yoga, classes are fully customizable, and can either be watched live, with real-time feedback from instructors, or accessed via an on-demand library (Hobbs, 2020). While consumers may not be able to leave their homes and in turn have fewer reasons to purchase new activewear, Mirror ensures that activewear remains integral to our daily lives, and foresees that many would want to look good while watching themselves in the exercise mirror. The liquid utopian theorization proposed here presents a useful explanatory framework which can be leveraged in future research exploring these new logics of retailing and experiential consumption that privilege immediacy, transience and hyper-individualization.

As a construct, utopia is also closely related to the notion of escape. To this end, contemporary liquid utopias shed new light on experiential consumption and the notion of escape from reality via consumption (see Cova et al., 2018). Seminally, consumption, and particularly experiential consumption, has been framed as an escape from the mundanity of the everyday (Holbrook & Hirschman, 1982). In this vein, Turner’s (1969) structure/anti-structure model has been applied widely in conceptualizing experiential consumption as positive, anti-structural and regenerative experiences that unfold in liminal spaces which challenge established structures and hierarchies. With their potential to bring immediate change, transformation and possibility (as compared to stasis, order, and structure), liquid consumer utopias echo Turner's description of the *communitas* (opposed to the *societas* demanding structure and order) where human spontaneity, self-constitution, experimentation, and transformation can freely unfold (Jacobsen, 2004). While holding much of the escapist potential found in anti-structural consumption, liquid consumer utopias, however, privilege individualization, not community, and the anti-structural and liminal is found in the hyper-personalized, not the shared. This is in agreement with Tumbat and Belk’s (2011) observation that extraordinary and anti-structural consumption experiences need not be conducive to feelings of community, but can be very individualistic and competitive (see also Husemann et al., 2016).

Liquid utopias offer opportunities for further problematizing the shifting boundaries of anti-structural consumption in contemporary modernity. Cova et al. (2018) advance the distinction between escapes “from” structures and escapes “into” anti-structures, and theorize experiential consumption according to the distance from home or the self that it facilitates. Future research can examine the new types of escape that liquid consumer utopias motivate, particularly in a time of unprecedented global uncertainty. For instance, Bauman (2007) proposes that liquid utopias are prompted by a desire to escape from present reality, not to run toward an idealized future. How does this notion blend with the imagining *and* the actualization of everyday escapes? With the future increasingly uncertain and everyday life fundamentally disrupted, how do consumers construct and materialize such utopian escapes from the confines of their homes?

As the consumer experience literature traditionally privileges Western subjectivity (Cova et al., 2018) there is also little empirical research on the plethora of escapist consumption unique for non-Western societies. Similar to the Chinese Sang culture referenced earlier, Japan’s rage rooms for de-stressing, are becoming a global phenomenon offering refuge from the everyday, in which women are punished for showing anger or where destruction is generally viewed to be against established norms (Brigita, 2017). A liquid utopian lens would suggest that such experiences are much more than mere releases of frustration, but quick and efficient re-imaginings of reality through short-term and emotionally charged experiential consumption that renders self-expression possible. Future research can better map the escapist and utopian potential of such consumption practices and further expand our understanding of the new forms of experiential consumption in liquidity.

Another fruitful avenue for research would be to explore the mechanisms that drive consumers’ orientations toward solid or liquid utopian modes of thinking––what are the various antecedents and consequences, on contextual and individual level, that frame consumers’ movement along the solid–liquid continuum (cf. Lamberton & Goldsmith, 2020)? Lamberton and Goldsmith (2020), for instance, infer that intrinsic and individual difference factors (e.g. psychographic, demographics, social trust) may combine with external factors (e.g. economic recession, uncertainty about the future) in predicting solid or liquid tendencies. The anecdotal evidence pertaining to utopian consumption presented earlier speaks to the plausibility of this effect. Future research will be well-positioned to theorize what factors influence consumers’ tendencies to adopt solid or liquid orientations in the process of envisioning a better way of being in the world. Essential for this line of research would also be to map out what accounts for an experience of liquid or solid utopia, or no utopia at all?

Finally, a liquid utopian perspective also sheds light on the growing domains of lifestyle design and self-optimization, which inherently focus on individuals’ aspirations for betterment within. For Bauman (1998), as for Giddens (1991, p.198), artificially framed styles of life and projects of self-actualization in liquidity are intensely commodified, packaged and distributed by the market in the form of self-help books. In liquidity, consumers are on the lookout for guides of living and blueprints for self-optimization, which are framed as means for “hacking life” (Ferriss, 2009) and dealing with the uncertainty felt by individuals in modern society (Bauman, 1998, p.178–179). These are essentially utopian pursuits for betterment in a life that is hard to control and filled with insecurities. In turn, a growing trend for consumption of the self (Rindfleish, 2005), guided by New Age spiritualities, have resulted in the proliferation of go-to tools, apps, techniques and books offering guidance not only for managing life, but mastering it and turning it into a triumphant utopian success (instantaneously at that). Research has shown that the physical body itself also holds potential for mediating utopian visions (Roux & Belk, 2019). Liquid utopias can thus be seen as productive avenues for arriving at such spiritual or bodily self-transformation. Also of interest would be to examine to what extent consumers’ aversion from future-planning and envisioning of the long-term affects their motivations to invest (themselves) in extended projects of identity building (such as those spelled out in the popular self-optimization literature), and how these tensions might be resolved through pursuit of liquid utopian projects.

## Conclusion

In this paper, we conceptualize the notion of liquid consumer utopias as market-mediated expressions of individuals’ desires to re-imagine and re-construct reality, and to re-frame the present. Drawing from contemporary utopian scholarship (Bauman, 2007; Levitas, 2011), we propose that unlike the solid, grand and collective consumer utopias of interest to prior research, liquid consumer utopias articulate a re-imagining of the present (rather than the future), have an emphasis on individual (rather than communal or collective) experiences of betterment, and an orientation toward temporary re-framings of the experienced reality (rather than a pursuit of permanence and long-lasting change). We demonstrate that solid and liquid consumer utopias can co-exist within today’s marketplace, and offer a theoretical lens that can shed light on emergent liquid forms of utopian consumption that have thus far been left out of sight. By conceptualizing the liquifying nature of the utopian imaginary, this work seeks to bring new conceptual energy to the construct of utopia within the marketing literature. To this end, we offer future research suggestions in three domains: retailing, experiential consumption, and consumer self-optimization. Overall, this paper offers a new lens toward decoding consumers’ contemporary desires for better living and being in an increasingly uncertain, unpredictable, and dematerialized world.
